# Correlation between the Rotation of the First Molars and the Severity of Class II Division 1 Malocclusion

**DOI:** 10.1155/2015/261485

**Published:** 2015-06-18

**Authors:** Betânia Pessoa Lima, Célia Regina Maio Pinzan-Vercelino, Laércio Santos Dias, Fausto Silva Bramante, Rudys Rodolfo De Jesus Tavarez

**Affiliations:** Ceuma University, Rua Josué Montello No. 1, Renascença II, 65075-120 São Luís, MA, Brazil

## Abstract

This study aimed to evaluate the potential correlation between the severity of Class II division 1 malocclusion and the magnitude of mesiopalatal rotation of the maxillary first molars. Scanned images of 104 cast models were grouped according to the severity of Class II malocclusion as follows: Group 1, 1/4 Class II malocclusion; Group 2, 1/2 Class II malocclusion; Group 3, 3/4 Class II malocclusion; and Group 4, complete Class II malocclusion. The rotation was measured using parameters described by Henry, Friel, and Ricketts, referred to as indicators 1, 2, and 3, respectively. The correlation was evaluated using the Spearman's correlation coefficient. The rotational indicators were compared using one-way analysis of variance. For all statistical analyses used *p* < 0.05, a positive correlation was observed between the severity of Class II malocclusion and the mesiopalatal rotation of the maxillary first molar. This correlation was statistically significant for indicator 1 between Groups 1 and 3 and for indicator 2 between Groups 1 and 4, which include cases of extreme malocclusion. In conclusion, there is a positive correlation between the severity of Class II division 1 malocclusion and the magnitude of mesiopalatal rotation in the maxillary first molars.

## 1. Introduction

Since the establishment of orthodontics, the occlusion of the first molars has been a major consideration in the diagnosis of malocclusion and in treatment planning. The importance of their position in assessing occlusion was first described by Angle, who considered the maxillary first molars the “key to occlusion” because they occupy a normal position more frequently than any other tooth and because of their anatomical location within a fixed bone structure (maxilla) relative to the skull base [1]. The first clear and simple definition of normal occlusion postulated that the mesiobuccal cusp of the maxillary first molar occluded in the buccal groove of the mandibular first molar [1]. However, this definition only described the anteroposterior molar relationship and did not consider other spatial axes.

Class II malocclusion is characterized by a poor sagittal relationship between the dental arches, with the lower arch relatively distal to the upper arch to varying degrees [2]. As a result, the buccal groove of the mandibular first molars is distally positioned when in occlusion with the mesiobuccal cusp of the maxillary first molar. When the maxillary teeth are positioned relatively anterior, the malocclusion is considered Class II division 1 [1, 3]. In these cases, the patients generally exhibit muscle imbalance with labial incompetence, convex profile, and pronounced overjet [4].

Patients with Class II malocclusion also have high prevalence of mesiopalatal rotation of the maxillary first molars, ranging from 83 to 95% [5, 6]. This is of great clinical relevance because the molar occlusal surface occupies a larger space on the arch as it rotates in the mesiopalatal direction owing to its trapezoidal shape [7, 8]. This hinders proper dental intercuspation [5, 7] and often worsens the altered anteroposterior relationship [7]. Thus, correct diagnosis and assessment of the severity of molar rotation in Class II malocclusion are important in the prognosis and are a determining factor in treatment selection [9]. Correcting the rotation allows the anteroposterior interarch discrepancy to be decreased, greatly simplifying the indicated treatment [10–12]. It should be noted that the severity of malocclusion is one important factor that must be considered in orthodontic treatment planning [9].

Although many studies have examined the prevalence of rotation in molar malocclusion Class II patients [5, 6, 13–16], few studies have examined the correlation between the severity of the anteroposterior molar relationship and the magnitude of rotation, which indicated the need for the present study. In this context, this study aimed to evaluate the correlation between the magnitude of rotation of the maxillary first molars and the severity of Class II malocclusion.

## 2. Methodology

This study was approved by the Ethics Committee in Research (protocol number 00255/11). We selected 104 dental casts from the initial orthodontic records of patients presenting with a Class II division I molar relationship. The sample size was calculated using a 5% alpha error and 80% power for a correlation coefficient of at least 0.20.

The orthodontic models were selected according to the following inclusion criteria: (1) absence of prior orthodontic treatment; (2) presence of bilateral Class II division 1 malocclusion; (3) presence of all erupted teeth; (4) absence of caries, fracture, proximal wear, or prosthetic restoration in the posterior teeth; (5) absence of significant dental anomalies in shape, size, and number; and (6) absence of cross-bites.

For sample selection, only the anteroposterior molar relationship in varying severities of Class II malocclusion was assessed. Due to the scarcity of models with equal severity bilaterally, we chose to assess each maxillary first molar individually. After sample selection, 208 first molars were included and divided into four groups according to the degree of anteroposterior discrepancy in the Class II molar relationship: (1) Group 1, 1/4 Class II molar relationship (*n* = 92); (2) Group 2, 1/2 Class II (*n* = 46); (3) Group 3, 3/4 Class II (*n* = 39); and (4) Group 4, complete Class II (*n* = 31). The discrepancy was determined according to the distance between the mesiovestibular groove of the lower first molar and the tip of the cusp of the mesiobuccal maxillary first molar as follows: 1/4 Class II, distance >1 mm and <3.5 mm; 1/2 Class II, distance = 3.5 mm; 3/4 Class II, distance >3.5 mm and <7 mm; and complete Class II, distance >7 mm [2].

**Figure 1:**
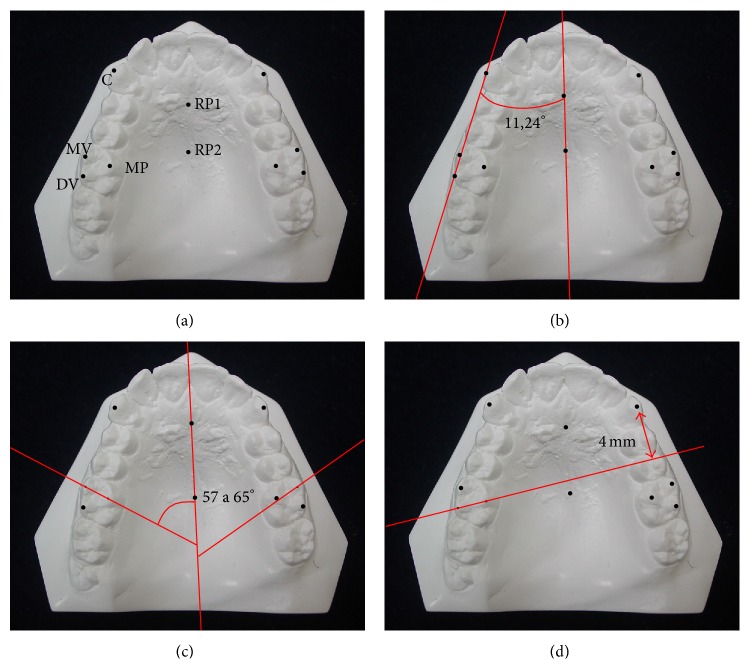
Determination of rotation of the first maxillary molar. (a) The defined points were initially marked on the scanned model. The three indicators were then calculated as follows: (b) indicator 1 (angle of Henry); (c) indicator 2 (angle of Friel); and (d) indicator 3 (line of Ricketts).

Six strategic points were marked initially on each model using an extra fine black brush (Figure 1(a)) to serve as a reference for the formation of the angles and lines, which were then used to evaluate the molar rotation. The various points, lines, and angles are described in Table 1.

The models were scanned at a 9600 × 4800 dpi resolution. Digital measurements were made using CorelDRAW X5 software (Corel, Ottawa, Canada), and the molar rotation was evaluated based on linear and angular indicators of molar rotation as follows:

1. Indicator 1 (angle of Henry): angle formed between the MV-DV line and RP1-RP2 line (Figure 1(b)); a molar with an angle of 11.24° was considered well positioned.
2. Indicator 2 (angle of Friel): angle formed between the palatine raphe and the MP-MV line (Figure 1(c)); values between 57° and 65° were considered normal.
3. Indicator 3 (line of Ricketts): the smallest distance between the DV-MP line and the tip of the cusp of the canine (C) on the opposite side (Figure 1(d)); the molar was considered well positioned at distances up to 4 mm.

Angle indicators 1 and 2 were proposed by Henry [5] and Friel [13] for evaluating first molar rotation and are justified because they are easily reproducible and have been validated [5, 8, 12, 13]. The line of Ricketts [17] (Indicator 3) was used because it is a traditional, linear, and easily applicable clinical measure. The indicators were measured in each individual molar because the study objective was not to quantitatively evaluate the molar rotation for each patient but rather to assess the magnitude of rotation according to the severity of Class II malocclusion in the anteroposterior molar relationship. Equal gender distribution between the groups was unnecessary because there are no statistically significant differences between women and men in the first molar rotation according to Dahlquist et al. [8]. The molars were evaluated individually by a single operator who marked the reference points and measured the indicators while blinded to the severity of Class II malocclusion in each molar.

The significance level was designated at 5% (*p* < 0.05) for all statistical analyses. To assess the intraexaminer error, the operator repeated the measurements 90 days after the first measurement, and 20% of the sample (41 molars) was randomly selected for analysis. The paired *t*-test was used to evaluate systematic errors, and the order of magnitude of casual errors was estimated using the formula described by Dalberhg [18].

The mean and standard deviation were calculated for each indicator of rotation in the four groups. The data normality was verified using the Kolmogorov–Smirnov test prior to statistical analysis. The correlation between Class II malocclusion severity and molar rotation indicators was assessed using the Spearman rank correlation coefficient. The mean of the rotational indicators was compared between the four groups using one-way analysis of variance (ANOVA), and the Tukey post hoc test for individual comparisons was performed when a statistically significant difference was detected.

## 3. Results

The paired *t*-test showed that there was no statistically significant difference between the two measurements performed by the same examiner (Table 2).

Table 3 summarizes the correlation between the severity of Class II malocclusion and the three indicators of molar rotation evaluated using the Spearman correlation coefficient. The results show a positive correlation between the severity of malocclusion and the mesiopalatal rotation of the molar for indicators 1 and 2 but showed no correlation when indicator 3 was evaluated.

Table 4 summarizes the comparison of all three indicators between the four groups using ANOVA complemented by the Tukey test. The analysis revealed a relationship between the severity of Class II malocclusion and the molar rotation for indicators 1 and 2 but revealed no association when indicator 3 was assessed. Regarding indicator 1, the Tukey test revealed a statistically significant difference between Groups 1 and 3 and between Groups 1 and 4. For indicator 2, only Groups 1 and 4 showed a statistically significant difference in the mesiopalatal rotation.

## 4. Discussion

The effectiveness of orthodontic treatment is closely related to proper therapeutic planning. In order to establish an effective treatment plan, attention must be paid to the detailed methods employed and diagnostic criteria [9]. Among various criteria to be considered, the positioning of the first permanent molars in the three spatial axes warrants attention. The anteroposterior relationship of these teeth is highly emphasized in previous reports; however, few studies [6, 11, 14, 19] have evaluated the first permanent molars in the transverse plane (occlusal view). Moreover, no previous study has examined the severity of Class II malocclusion in these teeth.

In this study, we observed a high mesial rotation in all groups, despite being at a lower prevalence than in previous studies [5, 6, 8, 14]. Lamons and Holmes [6] observed the most significant rotation in patients with early loss of the second deciduous molars. Currently, owing to improvements in conservative dental treatment and changes in oral hygiene habits, dental loss occurs less frequently, and, consequently, lower molar rotation can be observed.

The Spearman's rank correlation coefficient test revealed a positive correlation between the severity of Class II malocclusion and the magnitude of mesiopalatal rotation in the maxillary first molars when evaluated by indicators 1 and 2 (Table 3). The greater the severity of Class II malocclusion, the greater the mesiopalatal rotation of the molars, demonstrating contribution of rotation in the formation of malocclusion.

When the individual indicators were evaluated, the mean angle of Henry was 14.51°, which is very similar to the 14.98° found by Junqueira et al. [16] and close to the 15.3° found by Giuntini et al. [19], but less than the 17.38° found by Henry [5]. The mean angle of Friel was 58.25°, which is similar to the values reported by Giuntini et al. [19] and Junqueira et al. [16] (55.8° and 57.45°, resp.). Kanomi et al. [20] found a higher value for this indicator (63.5°), probably because their population comprised several malocclusion types and not Class II division 1 malocclusion alone.

It is noteworthy that the mean values of indicators 1 and 2 are very similar to those obtained in other studies [5, 16] of Class II malocclusion using a similar methodology. The small differences observed may result from other factors in addition to the malocclusion type that also causes rotation of the first molars, such as the upper arch morphology, changes in the shape and size of the maxillary first molars, canines, and premolars, malpositioning of the canines and premolars, the presence or absence of interproximal caries, and early loss of the deciduous molars.

Analysis with the ANOVA test revealed that these indicators were associated with the severity of Class II malocclusion. When each group was analyzed individually, we found that the magnitude of molar rotation was proportional to the severity of Class II malocclusion present. However, a statistically significant association was only found between Groups 1 and 3 and between Groups 1 and 4, suggesting that this relationship is more relevant at the extremes of Class II malocclusion (Table 4).

Indicator 3 (line of Ricketts) had a mean value of 14.77 mm in the present sample population. According to Ricketts [17], the first molar is well positioned when the line passing over the tip of the cusp of the canine is within 4 mm distal. The more distal this line, the greater the mesiopalatal rotation, while a more mesial line indicates greater distopalatal rotation. The mean value in this study indicates mesiopalatal rotation of the molar and was higher than the 11.38 mm value reported by Junqueira et al. [16] in Class II malocclusions. For this indicator, no correlation was observed between the severity of Class II malocclusion and the magnitude of molar rotation; however, values varied from 2.3 to 27.63 mm, indicating a high standard deviation in this measure. This large variation impedes the evaluation of molar rotation and the subsequent analyses using this indicator. Other factors, such as the shape of the dental arch, the anatomy of the first molar crown, and variation in the size and position of the canines and premolar crowns may explain these differences and warrant examination in future studies.

Given the importance of proper positioning of the maxillary first molar for establishing a normal occlusion and consequent stability after orthodontic treatment, the present study was designed and performed to verify that mesiopalatal molar rotation was potentially associated with the severity of Class II malocclusion.

## 5. Conclusion

Based on the present methodology and the results obtained, we concluded the following:

- There was a positive correlation between the severity of Class II division 1 malocclusion and the magnitude of mesiopalatal rotation of the maxillary first molars when measured using indicators 1 and 2 (angle of Henry and angle of Friel, resp.).
- This correlation was statistically significant for indicator 1 between Groups 1 and 3 and Groups 1 and 4, whereas for indicator 2, this correlation was statistically significant only in the most severe Class II malocclusion groups (Groups 1 and 4).

## Figures and Tables

**Table 1:** Points, lines, and angles used to evaluate molar rotation.

	Definition
Points	
RP1	Most anterior region of the palatine raphe
RP2	Most posterior region of the palatine raphe
MV	Tip of the mesiobuccal cusp of the maxillary first molar
DV	Tip of the distobuccal cusp of the maxillary first molar
MP	Tip of the mesiopalatal cusp of the maxillary first molar
C	Tip of the cusp of the maxillary canine

Lines	
Line MV-DV	Connection between points MV and DV
Line RP1-RP2	Connection between points RP1 and RP2
Line DV-MP	Connection between points DV and MP (line of Ricketts)

Angles	
Angle of Henry	Angle formed between MV-DV and RP1-RP2 lines
Angle of Friel	Angle formed between the palatine raphe and the MP-MV line

**Table 2:** Mean, standard deviation, statistical significance, and error for assessment of interexaminer error.

Indicator	Mean 1	Mean 2	SD 1	SD 2		*p*	Error
1	15.38 mm	15.59 mm	7.52	7.51	−0.94	0.34	0.97
2	60.95 mm	60.71 mm	7.69	7.75	1.45	0.15	0.76
3	15.03°	15.17°	4.23	4.07	—	0.19	0.48

SD: standard deviation; rotation in the first maxillary molar was measured initially (mean 1, SD 1) and 90 days later (mean 2, SD 2) by a single examiner.

**Table 3:** Spearman correlation analysis of the relationship between the severity of Class II malocclusion and the molar rotation.

Correlation	*R*	*p*
Severity of Class II malocclusion × indicator 1	0.26	<0.001^*∗*^
Severity of Class II malocclusion × indicator 2	−0.17	0.013^*∗*^
Severity of Class II malocclusion × indicator 3	0.13	0.058

^*∗*^Significant correlation designated at *p* < 0.05.

**Table 4:** Mean, standard deviation, and comparison between molar rotation indicators in the four Class II malocclusion groups.

Group	Indicator 1	Indicator 2	Indicator 3
1	12.60 ± 6.17^a^	59.96 ± 6.96^c^	13.90 ± 4.49^e^
2	15.21 ± 5.87^ab^	57.62 ± 7.47^cd^	15.89 ± 4.58^e^
3	16.43 ± 7.51^b^	56.71 ± 7.94^cd^	15.35 ± 4.22^e^
4	16.77 ± 7.05^b^	56.05 ± 9.85^d^	15.01 ± 4.42^e^

Data are presented as the mean ± standard deviation. Indicators 1, 2, and 3 (angle of Henry, angle of Friel, and line of Ricketts, resp.) were compared between the four groups using analysis of variance (ANOVA). Different letters represent significant differences (Tukey's test).
